# Geospatial measurement of urban sprawl using multi-temporal datasets from 1991 to 2021: case studies of four Indian medium-sized cities

**DOI:** 10.1007/s10661-022-10542-6

**Published:** 2022-10-10

**Authors:** Vishal Chettry

**Affiliations:** Manipal School of Architecture & Planning, Manipal Academy of Higher Education, Manipal, India

**Keywords:** Urban sprawl, Relative entropy, Geospatial techniques, Medium-sized city, India

## Abstract

In recent decades, medium-sized Indian cities have experienced accelerated urban growth due to the saturation of large cities. Such rapid urban growth combined with inadequate urban planning has triggered urban sprawl in medium-sized Indian cities. In this context, the present study focuses on the geospatial measurement of urban sprawl in four rapidly expanding Indian medium-sized cities located in diverse physiographic regions, such as Lucknow urban agglomeration (UA), Bhubaneswar UA, Raipur UA, and Dehradun UA. Multi-temporal Landsat imageries from 1991 to 2021 were downloaded for land cover classification through the maximum likelihood classification tool in ArcGIS 10.3. Thereafter, spatiotemporal land cover change detection was performed based on the classified land cover maps. The presence of urban sprawl was detected using the relative entropy index while the urban expansion index quantified the urban sprawl typologies such as edge expansion, leapfrog development, and ribbon development. The results exhibited a rapid rise in built-up land cover from 1991 to 2021. The prevalence of urban sprawl was detected in all four cities as per the relative entropy index. Edge expansion typology of urban sprawl was dominant compared to leapfrog development and ribbon development. Such urban growth phenomenon creates a hindrance in promoting sustainable urban development in medium-sized Indian cities. The results obtained from this paper would assist urban planners and policymakers in developing strategies to encourage planned urban growth. This paper exhibits the potential of geoinformatics to monitor and analyze urban sprawl.

## Introduction

Rapid urbanization and the expansion of cities are two of the most significant worldwide phenomena noticed this century (World Economic Forum, 2015, 2016, 2017). Compared to 1950, when just 30% of the global population was urbanized, cities now house more than half (54% in 2014) of the global population (United Nations, 2015a). Clark (1982) argues that urbanization is a spatial and demographic process wherein a settlement or village transforms its basic characteristics into a town or city. Globally, urbanization occurred primarily as a result of the conversion from rural to urban areas, or due to the influx of population into existing cities (Yildiz & Doker, 2016; Lima & Romanelli, 2019).

The areas undergoing rapid urbanization have some typical characteristics such as accelerated urban activities and decline in rural activities; rapid and haphazard development with insufficient infrastructure; loss of prime agricultural lands, forests, surface, and underground water bodies; mostly habituated by middle and low-income residents; vulnerable to environmental damage dominated by landfill sites and industries; and also attracts rental market (Salvati, 2016; Jamali & Kalkhajeh, 2019). Overall such transformation in the last few decades has promoted urban sprawl and degraded the natural environment, thus challenging sustainable urban development (Bihamta et al., 2015; Meer et al., 2021).

Urban sprawl lacks an universally recognized definition due to the variation in characteristics among developed and developing nations (Nazarnia et al., 2019). The term urban sprawl was first mentioned by Earle Draper in his speech at a national conference of planners in 1937 to describe its undesirable effects on society and the economy (Wassmer, 2002). Post-1961, the concept of urban sprawl gained momentum after Jane Jacobs published her famous essay, “The death and life of great American cities.” As per Zhang (2004), in the mid-twentieth century, the urbanized area in the USA extended outwards rapidly through the conversion of farmlands and forests into urban uses. However, a widely accepted notion of urban sprawl represents separated land use, scattered development, horizontal and unplanned growth, and poor environment which ultimately promotes improper utilization of land (Doygun, 2009; Kumar et al., 2010; Sallustio et al., 2016; Varol et al., 2019). As a consequence, many Indian cities, such as Delhi, Ajmer, Greater Bangalore, Kolkata, and Nagpur, are experiencing urban sprawl (Sokhi et al., 1989; Jat et al., 2008a, b; Ramachandra et al., 2012; Dutta et al., 2015; Mithun et al., 2016; Kar et al., 2018). Hence, it is important to spatially measure the temporal urban sprawl for avoiding unplanned urban growth (Ziegler, 2009; Silvia et al., 2011).

Among the Indian studies, the researchers have analyzed urban sprawl by focusing on various factors, which include, but are not limited to, land cover change detection (Vani & Prasad, 2020), increase in impervious land surface (Chettry & Manisha, 2022), loss of natural land covers and ecological devastation (Poyil & Misra, 2015), and rise of urban heat island. Remote sensing (RS) datasets combined with geographic information system (GIS) techniques are efficiently utilized to investigate the dynamics of urban growth and identify urban sprawl (Navalgund et al., 1996; Kumar et al., 2017; Chettry, 2022). It is a cost-effective and an efficient tool for the research as it provides satellite images from the different period, covers a vast area, and provides the feasibility of automation, which is required in the research. The entropy indexes, such as structure entropy, Shannon’s entropy, and relative entropy, combined with RS and GIS were attempted in several studies (Jiang et al., 2016; Xu et al., 2018). Harvey and Clark (1965) classified the patterns of urban sprawl into three major categories, i.e., continuous low-density development, ribbon development, and leap-frog development. Wilson et al. (2003) categorized urban growth into three categories, i.e., infill development, edge expansion, and outlying growth. Landscape metrics are calculated using the open-source software package FRAGSTATS v4.2.1 to examine the urban sprawl characteristics (Sarif & Gupta, 2021). Furthermore, the typologies of urban sprawl such as secondary urban core, urban fringe, ribbon development, and scatter development are identified through the adjacent neighborhood relationship concept (Chettry & Surawar, 2020).

In India, the large cities (population of 5 million or more) have surpassed their carrying capacity and intense built-up growth is forecasted in medium-sized cities, i.e., population between 0.5 and 5 million. Such forecasted rapid growth in Indian medium-sized cities combined with lack of proper urban planning would induce unplanned urban growth and negatively impact the local environment (Nandi & Gamkhar, 2013). Moreover, medium-sized cities are suggested to be the center of balanced regional development; however, they have been sidelined in developing countries (United Nations, 2013). On the contrary, the major focus has been mostly on large cities, such as Delhi (Kumar et al., 2022), Mumbai (Shahfahad et al., 2021), Kolkata (Rukhsana & Hasnine, 2021), Chennai (Sridhar & Sathyanathan, 2022), Bengaluru (Biswas et al., 2022), Hyderabad (Hatab et al., 2021), Ahmedabad (Mohammad et al., 2019), and Pune (Kantakumar et al., 2020). Overall, the research focused on the urban sprawl assessment in Indian medium-sized cities is relatively scarce (Chettry, 2022).

United Nations in 2015 adopted the 2030 Agenda for Sustainable Development, wherein goal no. 11 promotes sustainable cities and communities through the practice of sustainable urban planning (United Nations 2015b). Hence, it is crucial to monitor and quantify the occurrence of spatiotemporal urban sprawl in Indian medium-sized cities. Therefore, this paper focuses on the analysis of urban sprawl patterns in four Indian medium-sized cities located in diverse physiographic regions from 1991 to 2021. Urban agglomeration (UA) defined by the Census of India is selected as a unit of investigation in this study. This administrative unit is most appropriate because it encompasses the nearby outgrowths and census towns, where urban sprawl is likely to occur (Ghosh & Das, 2017). In the following section of this research paper, the “Study area and datasets” section defines the study areas and highlights datasets, the “Research methods” section focuses on the overall methodology and the methods, the “Results” section presents the results, the “Discussions” section discusses the results, and the “Conclusion” section exhibits the conclusion.

## Study area and datasets

**Fig. 1:**
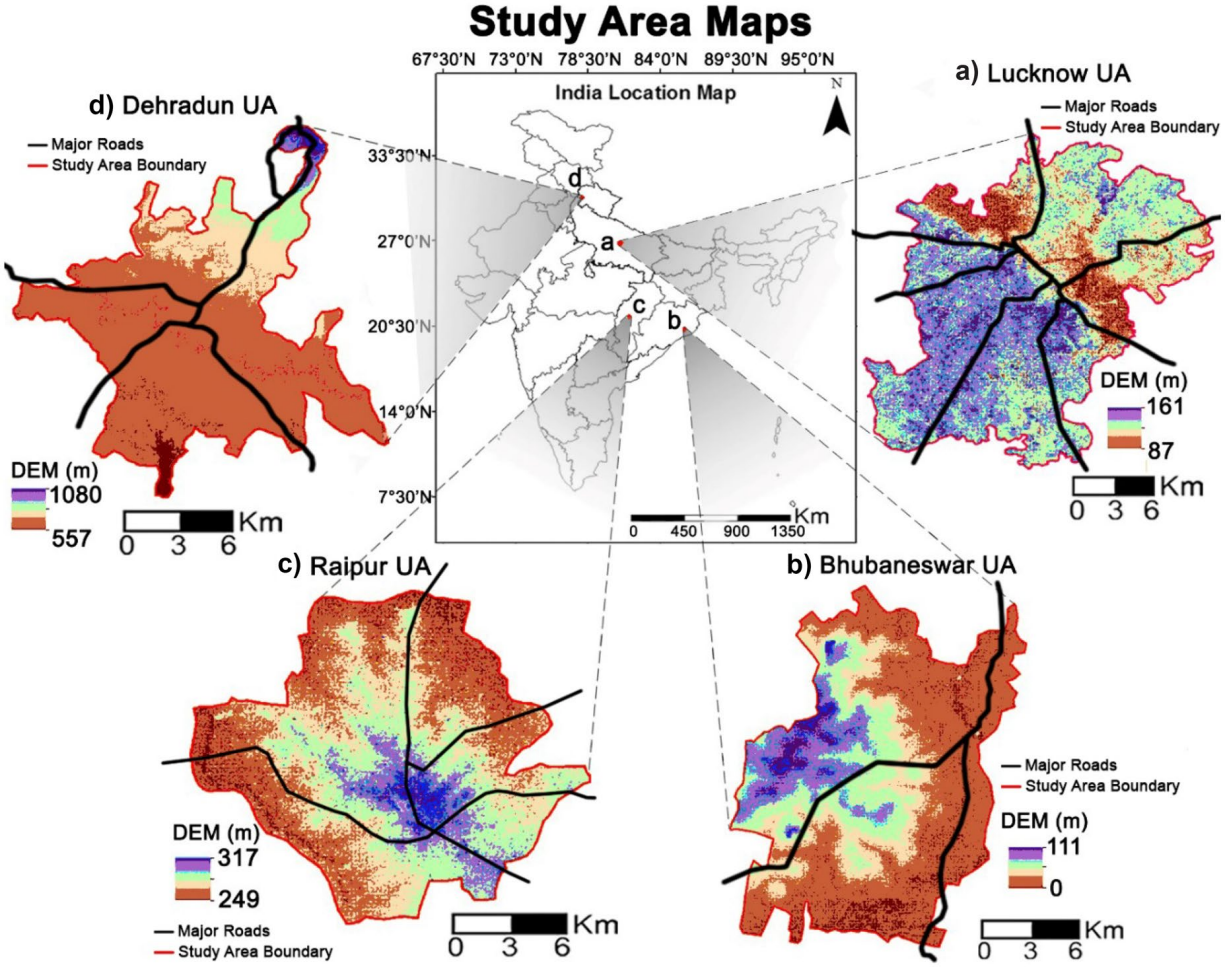
Study area maps. **a **Lucknow UA. **b **Bhubaneswar UA. **c **Raipur UA. **d **Dehradun UA

The four medium-sized cities selected in this paper for the investigation of urban sprawl are Lucknow UA from Indo-Gangetic Plains, Bhubaneswar UA from Coastal Plains, Raipur UA from Peninsular Plateaus, and Dehradun UA from Northern Mountains (Fig. 1).

Lucknow is the capital city of Uttar Pradesh state of India and the administrative headquarters of the Lucknow district. The total area of Lucknow UA is 393.60 km^2^ and it includes Lucknow Municipal Corporation, one cantonment board, and two census towns. The city of Raipur is a capital city of Chhattisgarh state of India and an administrative headquarters of the Raipur district. Raipur UA encompasses an area of 192.55 km^2^ and consists of Raipur Municipal Corporation, two outgrowths, and one municipality. Bhubaneswar is the capital city of Odisha state and is famously known as the “Temple City of India.” Bhubaneswar UA encompasses an area of 185.30 km^2^ and comprises of Bhubaneswar Municipal Corporation and fourteen outgrowths. Dehradun is the capital city of Uttarakhand state of India and the administrative headquarters of the Dehradun district. Dehradun UA encompasses an area of 125.22 km^2^ and includes Dehradun Municipal Corporation, two cantonment boards, three census towns, and one outgrowth.

The details about spatial expanse of the study areas, i.e., details of UA, were obtained from the Census of India 2011 website. Landsat images for the years 1991, 2001, 2011, and 2021 were downloaded from the EarthExplorer United States of Geological Survey (USGS) website to analyze urban sprawl. The satellite images with < 20% cloud cover were selected for this research. The UA boundary for this study was not readily available; therefore, it was digitized and georeferenced in ArcGIS 10.3 with the help of Google Earth features like national and state highways. Microsoft Excel 2017 and ArcGIS 10.3 were used for analysis and data representation. Table 1 presents detailed information about each Landsat image used in this study.

**Table 1:** Description of Landsat satellite images used in this study

**S. no**	**Study areas**	**Acquisition date (yyyy-mm-dd)**	**Landsat sensor**	**Path/row**	**No. of bands**	**Grid cell size (m)**
1	Lucknow UA	1991–03-16	5 TM	144/41	7	30
		2001–03-03	7 ETM		9	
		2011–03-07	5 TM		7	
		2021–03-02	8 OLI TIRS		11	
2	Bhubaneswar UA	1991–01-08	5 TM	139/46	7	30
		2001–01-19	5 TM		7	
		2011–01-22	5 TM		7	
		2021–03-15	8 OLI TIRS		11	
3	Raipur UA	1991–01-29	5 TM	142/45	7	30
		2001–02-09	5 TM		7	
		2011–01-04	5 TM		7	
		2021–03-04	8 OLI TIRS		11	
4	Dehradun UA	1991–01-09	5 TM	146/39	7	30
		2001–01-20	5 TM		7	
		2011–02-01	5 TM		7	
		2021–03-16	8 OLI TIRS		11	

## Research methods

**Fig. 2:**
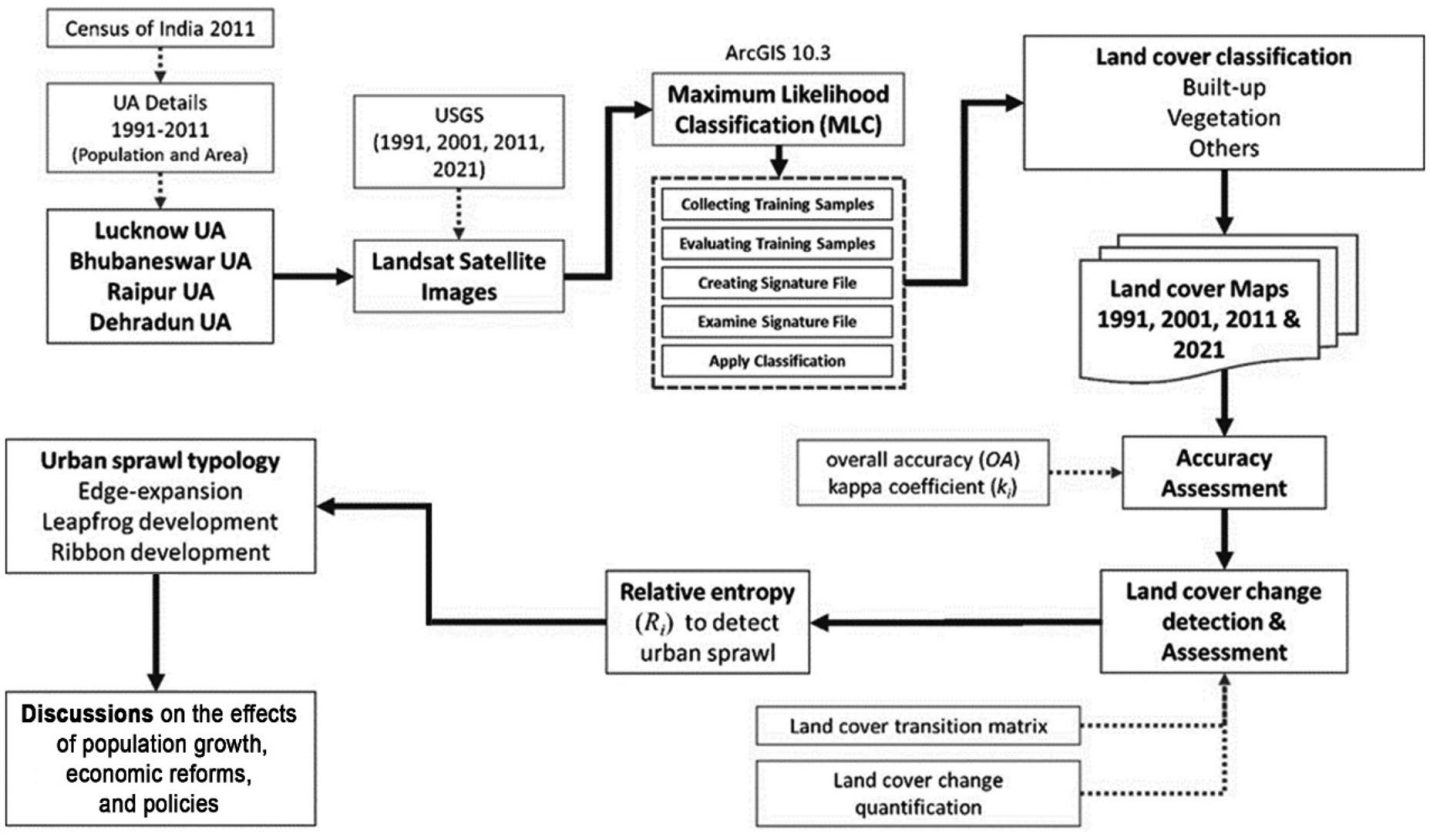
Overall methodology adopted in this study

The overall methodology adopted in this study is exhibited in Fig. 2. The downloaded Landsat satellite images (5TM, 7ETM, and 8OLI_TIRS) of the years 1991, 2001, 2011, and 2021 were utilized to perform spatiotemporal land cover change detection. The satellite images were pre-processed in ArcGIS 10.3, and the supervised maximum likelihood classification tool was employed to produce land cover maps. The land cover of the study areas was classified into three major categories, i.e., built-up, vegetation, and others. The accuracy of the classified land cover maps was assessed through indexes such as overall accuracy and kappa coefficient (Chamling & Bera, 2020). After obtaining satisfactory accuracy results, land cover change detection during each duration (1991–2001, 2001–2011, and 2011–2021) was quantified. The “from-to” changes within land covers, i.e., conversion of vegetation and other land cover category, to built-up was measured using the transition matrix. Thereafter, relative entropy index was used to detect urban sprawl in the four study areas. Urban expansion index was employed to quantify the typologies of urban sprawl from 1991 to 2021. Furthermore, the effects of population growth, economic reforms, and policies on the urban growth pattern were discussed.

### Land cover classification and accuracy assessment

1 $$P\left(A|B\right)=\frac{P\left(B|A\right)P\left(A\right)}{P\left(B\right)}$$

where $A$ and $B$ are events and $P\left(B\right)\ne$ 0; $P\left(A|B\right)$ is the likelihood of event $A$ occurring given that $B$ is true, $P\left(B|A\right)$ is the likelihood of event $B$ occurring given that $A$ is true, and $P\left(A\right)$ and $P\left(B\right)$ are the probabilities of observing $A$ and $B$ independently of each other.

The spectral tool of the ArcGIS 10.3 was used to merge the multiple bands of Landsat satellite images. The combined temporal imageries were georeferenced to Universal Transverse Mercator (UTM) Zone 44 N for Lucknow UA, UTM 45 N for Bhubaneswar UA, and UTM 44 N for Raipur UA and Dehradun UA. The preprocessing methods of the Landsat images comprised of spectral enhancement and radiometric normalization that includes noise removal, smoothing, and sharpening of images. The atmospheric correction of the satellite images was done using the dark-object subtraction (DOS) method (Dutta & Das, 2019). The Landsat images were classified into three major classes, i.e., vegetation, built-up, and other land cover categories based on the scope of this study (Aburas et al., 2018). The vegetation land cover includes forests, shrubs, lawns, and tree cover; built-up land cover includes all man-made structures, including transportation facilities and networks; and other land cover category includes waterbody, agricultural lands, fallow land, and open land. The maximum likelihood supervised classification (MLC) method in ArcGIS 10.3 was used to perform land cover classification in the study area during 1991, 2001, 2011, and 2021 (Mehdipour et al., 2019). This method is based on Bayes’ theorem (Eq. 1) to predict pixel probability for the highest likelihood (Alkaradaghi et al., 2019). In this method, 50 training samples for each land cover category were collected from the raster datasets (Almazroui et al., 2017). Thereafter, the collected samples were evaluated before creating a signature file. Based on these steps, the Landsat images were classified into three land cover categories for the study period in ArcGIS 10.3. Despite these methods, a few instances of mixed pixels discovered during the land cover classification procedure were eliminated through the reclassification process. The accuracy assessment of all the temporal land cover maps was evaluated using ground truth data and Google Earth archives (Ramachandra et al., 2014). This process is required to assess the accuracy of the land cover maps generated through the MLC process. Fifty equalized random ground truth points for each land cover class, i.e., a total of 150 sample points, were compared with the actual landscape data obtained through historical imagery in Google Earth Pro. The indexes employed to determine accuracy standards are overall accuracy (OA) and kappa coefficient (*k*_*i*_) as shown in Eqs. (2) and (3) (Chettry & Surawar, 2021).

2 $$\mathrm{OA}=\frac{\sum_{i=1}^{k}{n}_{ij}}{n}$$

3 $${k}_{i}=\frac{{P}_{(\mathrm{o})}- {P}_{(\mathrm{e})} }{1- {P}_{(\mathrm{e})}}$$

where ${n}_{ij}$ is the diagonal elements in the error matrix, $k$ is the total number of classes, $n$ is the total number of samples in the error matrix, ${P}_{(\mathrm{o})}$ is the observed proportion of agreement, and ${P}_{(\mathrm{e})}$ is the proportion expected by chance.

Thereafter, land cover change detection was performed to analyze the transformation of land covers in the four study areas during 1991–2021 (Raval & Shamsoddini, 2014).

### Land cover transition matrix

The land cover transition matrix often referred as cross-tabulation matrix was used to explain the nature of change in land covers using various spatiotemporal land cover datasets (Moghadam & Helbich, 2013; Haque & Basak, 2017). This approach is used to spot changes in land cover by image-to-image comparison of different periods (Shahbazian et al., 2019). In this research, the contribution of other land cover categories to built-up land cover between pre-date and post-date was identified using the land cover transition matrix (Zhao et al., 2014). This method was performed in the ArcGIS software to compute “from-to” (class $i$ to *j*) change in each pixel of the land cover categories Eq. (4).

4 $${P}_{ij}=\left[\begin{array}{ccc}{p}_{11}& \dots & {p}_{1n}\\ \vdots & \vdots & \vdots \\ {p}_{n1}& \dots & {p}_{nn}\end{array}\right]$$

where ${P}_{ij}$ reveals the amount of the landscape that experienced a transition from class $i$ to class $j$ between ${t}_{1}$ and ${t}_{2}$, and $n$ is the total number of land cover types.

### Relative entropy index

The built-up land cover was extracted from the land cover maps to analyze the patterns of urban growth in the study areas. Relative entropy index (*R*_*i*_) based on the Shannon’s entropy index was used to detect urban sprawl, as shown in Eqs. (5)–(7) (Gupta et al., 2018; Mestri et al., 2020). It highlights the amount of likely extreme dispersion in which a variable is spread between categories or spatial zones (Bagheri & Tousi, 2018). The study areas were divided into eight cardinal zones to determine the values. The entropy values range between 0 and 1, wherein values closer to 0 indicate compact development, while values closer to 1 indicate the prevalence of urban sprawl.

5 $${H}_{n}=-{\sum }_{i=1}^{n}Pi \mathrm{loge}(Pi)$$

6 $${P}_{i}={X}_{i}/\sum_{i=1}^{n}{X}_{i}$$

7 $${R}_{i}={H}_{n}/\mathrm{loge}(n)$$

where *P*_*i*_ is the possibility of the variable occurring in the zone *i*. In this research, the study area is divided into eight cardinal directions; therefore, the value of *n* is 8.

### Urban expansion index

Urban expansion index (UEI) quantifies the urban sprawl typologies observed in the four study areas (Shukla & Jain, 2019). This method quantitatively categorizes the development pattern of recent built-up patches surrounding the past developments. The distance between the existing developed areas and newly developed built-up patches is crucial to identify the urban sprawl typology. UEI is the ratio between the length of a common boundary between past and recent built-up patches and the patch perimeter of the recent built-up patches Eq. (8).

8 $$\mathrm{UEI}=\frac{{l}_{p}}{P}$$

where UEI is the urban expansion index, *l*_*p*_ is the length of the common edge between a new and old urban patch, and *P* is the perimeter of the new urban patch.

**Fig. 3:**
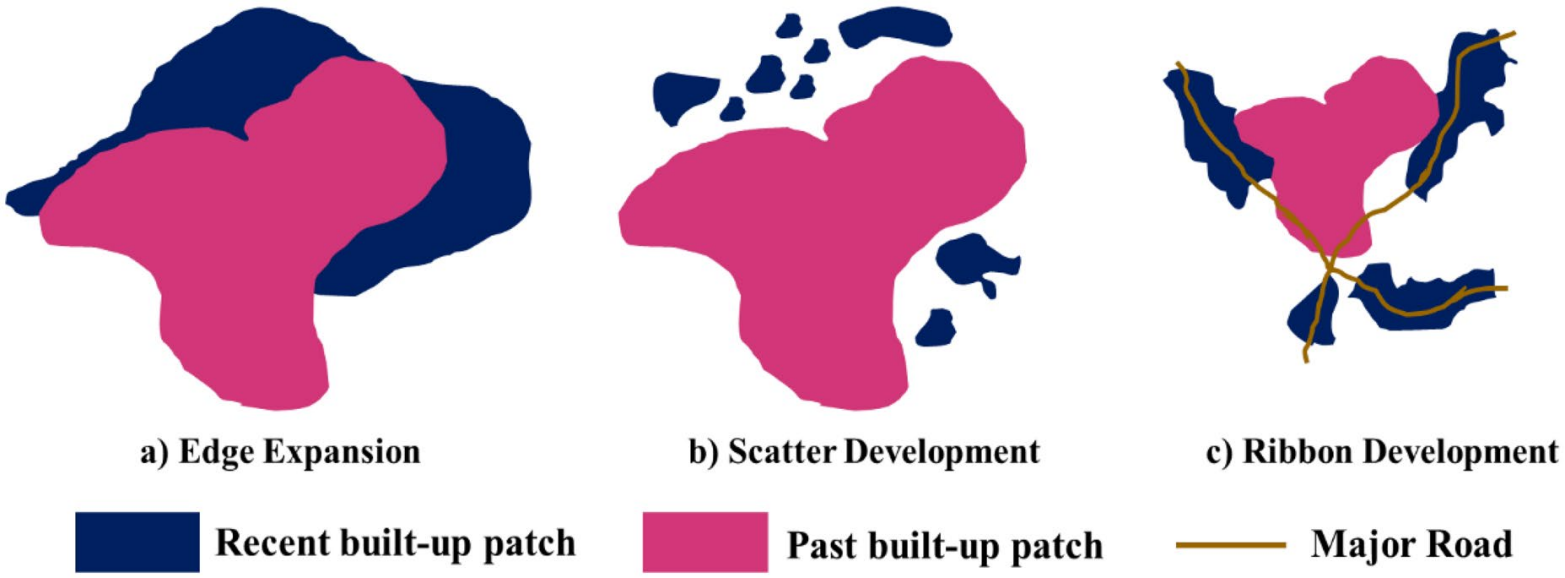
Urban sprawl typologies as per Wilson et al. (2003)

UEI quantifies the built-up growth pattern into three categories such as edge expansion (0 < UEI < 0.5), scatter development (UEI = 0), and ribbon development (0.5 < UEI < 0.75) (Wu et al., 2016). Edge expansion patches are the new built-up patches formed as an extension of existing built-up patches. Scatter development occurs independently and without any overlap to the existing built-up patches. Ribbon development occurs along the 100 m proximity of major roads within an urban area. The major roads were identified as the national highway and state highways passing through the city (Fig. 3).

## Results

### Land cover classification and change detection

**Fig. 4:**
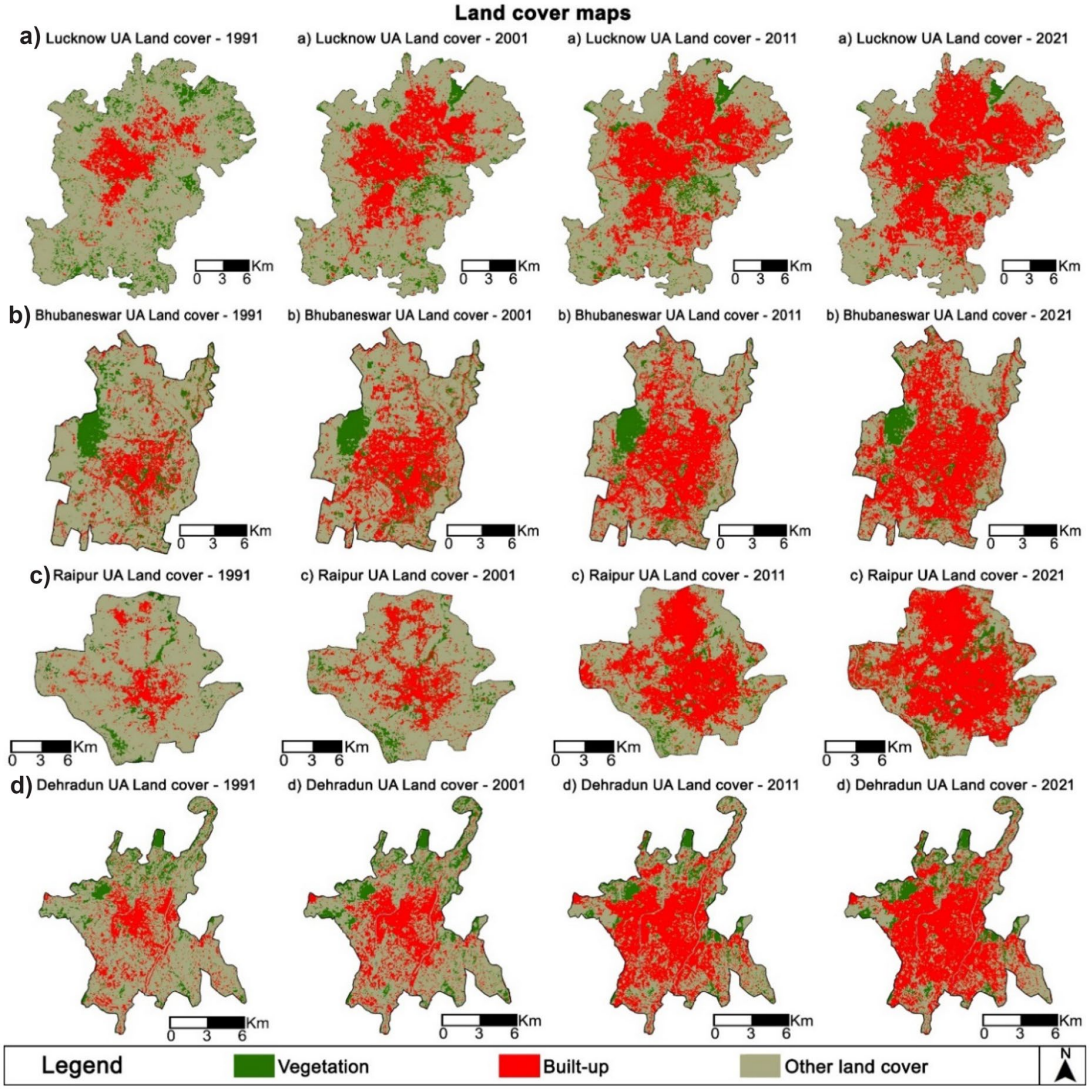
Land cover maps from 1991 to 2021. **a** Lucknow UA. **b **Bhubaneswar UA. **c **Raipur UA. **d **Dehradun UA

Figure 4 exhibits the land cover maps of four study areas during 1991, 2001, 2011, and 2021 obtained through the MLC method in ArcGIS 10.3. The accuracy assessment of the classified land cover maps is presented in Table 2. The results revealed overall accuracy (OA) and kappa coefficients (${k}_{i}$) above 85% and found to be satisfactory for further analysis (Saadani et al., 2020).

**Table 2:** Accuracy assessment of the land cover maps (1991, 2001, 2011, and 2021)

**S. no**	**Study areas**	**Overall accuracy (OA)**	**Kappa coefficient** **(k**_**i**_**)**
1	Lucknow UA	91.76–89.45%	88.27–85.49%
2	Bhubaneswar UA	93.61–91.27%	90.51–88.35%
3	Raipur UA	92.83–90.46%	89.79–87.64%
4	Dehradun UA	91.58–89.83%	88.94–86.72%

**Fig. 5:**
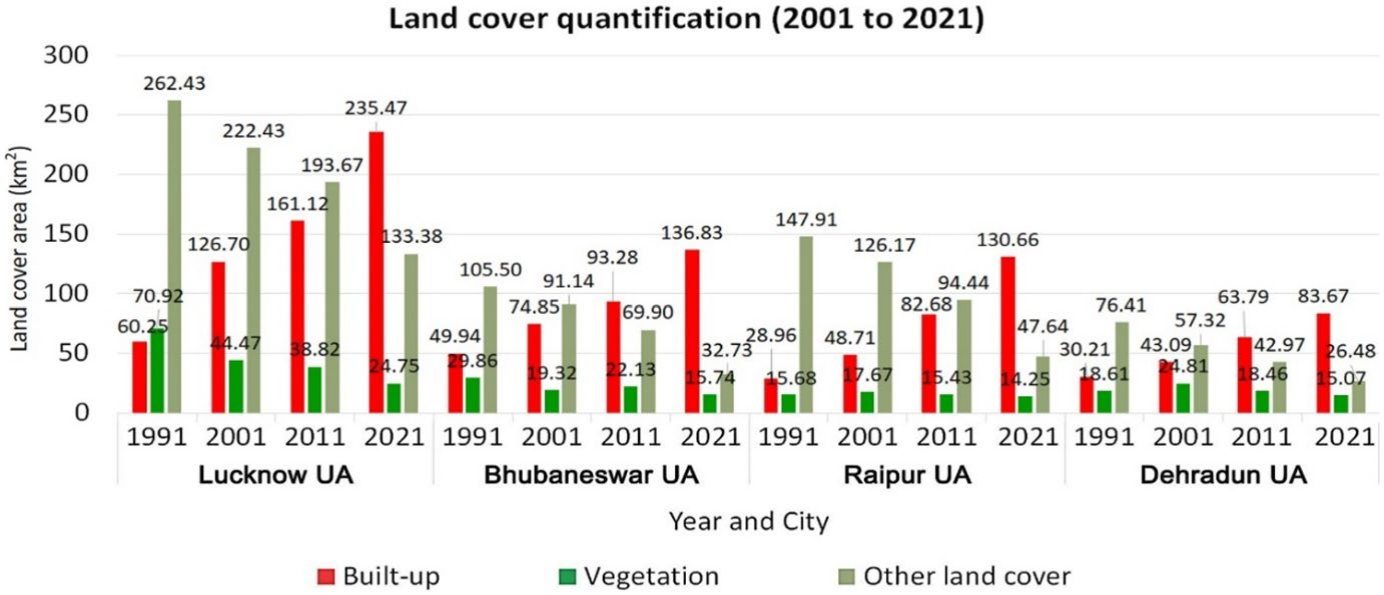
Land cover area details of four study areas during 1991, 2001, 2011, and 2021

The land cover of the four study areas was quantified and presented in Fig. 5. In Lucknow UA, the built-up land cover had increased from 60.25 km^2^ (15%) in 1991 to 235.47 km^2^ (60%) in 2021, with a growth rate of 291%. Vegetation land cover declined from 70.92 km^2^ (18%) in 1991 to 24.75 km^2^ (6%) in 2021, with a rate of −65%. Other land covers also declined from 262.43 km^2^ (67%) in 1991 to 133.38 km^2^ (34%) in 2021, with a rate of −49%. A similar trend was observed in Bhubaneswar UA, wherein built-up land cover had increased from 49.94 km^2^ (27%) in 1991 to 136.83 km^2^ (74%) in 2021, with a growth rate of 174%. Vegetation land cover declined from 29.86 km^2^ (16%) in 1991 to 15.74 km^2^ (8%) in 2021, with a rate of −47%. Other land covers also declined from 105.50 km^2^ (57%) in 1991 to 32.73 km^2^ (18%) in 2021, with a rate of −69%.

In Raipur UA, the built-up land cover had increased from 28.96 km^2^ (15%) in 1991 to 130.66 km^2^ (68%) in 2021, with a growth rate of 351%. Vegetation land cover also exhibited minimal increase from 15.68 km^2^ (7%) in 1991 to 14.25 km^2^ (7%) in 2021, with a rate of −9%. Other land covers declined from 147.91 km^2^ (77%) in 1991 to 47.64 km^2^ (25%) in 2021, with a rate of −68%. Lastly, in Dehradun UA, built-up had increased from 30.21 km^2^ (24%) in 1991 to 83.67 km^2^ (67%) in 2021, with a growth rate of 177%. Vegetation land cover declined from 18.61 km^2^ (15%) in 1991 to 15.07 km^2^ (12%) in 2021, with a rate of −19%. Other land covers also declined from 76.41 km^2^ (61%) in 1991 to 26.48 km^2^ (21%) in 2021, with a rate of −65%. Overall, maximum growth rate of built-up land cover was observed in Raipur UA (351%) compared to other selected study areas. Vegetation land cover exhibited a declining trend from 1991 to 2021 in all the four study areas.

The land cover transition matrix explains the pattern of land cover change to built-up using various spatiotemporal land cover datasets during 1991–2001, 2001–2011, and 2011–2021 (Table 3). Overall, significant transition from other land cover category to built-up land cover had occurred during the study period in all the four study areas.

**Table 3:** Land cover transition to built-up category

**Land cover classes**	**Lucknow UA**			**Bhubaneswar UA**		
	**1991–2001**	**2001–2011**	**2011–2021**	**1991–2001**	**2001–2011**	**2011–2021**
**Vegetation**	6.70%	4.07%	2.70%	4.69%	7.15%	8.16%
**Other**	63.40%	58.07%	49.57%	68.93%	59.52%	47.65%
**Land cover classes**	**Raipur UA**			**Dehradun UA**		
	**1991–2001**	**2001–2011**	**2011–2021**	**1991–2001**	**2001–2011**	**2011–2021**
**Vegetation**	1.63%	2.68%	3.65%	6.16%	8.26%	2.87%
**Other**	64.81%	57.35%	41.89%	68.31%	60.06%	64.62%

### Detection of urban sprawl and typologies

The occurrence of urban sprawl was observed in all four cities through relative entropy index (*R*_*i*_) (Table 4). In Lucknow UA, *R*_*i*_ values increased from 0.89 in 1991 to 0.99 in 2021, in Bhubaneswar UA from 0.91 in 1991 to 0.97 in 2021, in Raipur UA from 0.93 in 1991 to 0.98 in 2021, and lastly in Dehradun UA from 0.82 in 1991 to 0.93 in 2021. The entropy values in all the cities were approaching towards 1, which suggests the prevalence of urban sprawl. Moreover, entropy index exhibited an increasing trend in all four cities, thus indicating a rise in urban sprawl. There was variation in the pattern of entropy values in all the four cities during each year mostly, due to differences in the built-up growth pattern.

**Table 4:** Relative entropy index values in study areas

**S. no**	**Study areas**	**Relative entropy index** **(*R***_***i***_**)**			
		**1991**	**2001**	**2011**	**2021**
**1**	Lucknow UA	0.89	0.95	0.98	0.99
**2**	Bhubaneswar UA	0.91	0.92	0.96	0.97
**3**	Raipur UA	0.93	0.95	0.97	0.98
**4**	Dehradun UA	0.82	0.88	0.90	0.93

Overall, Lucknow UA was highly affected by urban sprawl followed by Raipur UA, Bhubaneswar UA, and Dehradun UA. Rapid and haphazard built-up growth is the main driving factor for urban sprawl rise in all the selected cities. Moreover, the low floor space index (FSI) or floor area ratio (FAR) has aggravated poor land utilization practices in all the four medium-sized cities.

The urban sprawl typologies (UST) in the four study areas from 1991 to 2021 are quantified and displayed in Table 5. In Lucknow UA, the edge expansion increased from 62% during 1991–2001 to 69% during 2011–2021, scatter development increased from 12% during 1991–2001 to 20% during 2011–2021, and ribbon development also increased from 2% during 1991–2001to 6% during 2011–2021. In Bhubaneswar UA, the edge expansion increased from 56% during 1991–2001 to 65% during 2011–2021, scatter development increased from 10% during 1991–2001 to 18% during 2011–2021, and ribbon development also increased from 1% during 1991–2001 to 3% during 2011–2021.

**Table 5:** Urban sprawl typologies in study areas

**S. no**	**Study areas**	**Urban sprawl typologies (UST) from 1991 to 2021**		
		**Edge expansion**	**Scatter development**	**Ribbon development**
**1**	Lucknow UA	62–69%	12–20%	2–6%
**2**	Bhubaneswar UA	56–65%	10–18%	1–3%
**3**	Raipur UA	62–66%	14–18%	2–3%
**4**	Dehradun UA	58–64%	13–24%	5–8%

In Raipur UA, the edge expansion increased from 62% during 1991–2001 to 66% during 2011–2021, scatter development increased from 14% during 1991–2001 to 18% during 2011–2021, and ribbon development also increased from 2% during 1991–2001 to 3% during 2011–2021. In Dehradun UA, the edge expansion increased from 58% during 1991–2001 to 64% during 2011–2021, scatter development increased from 13% during 1991–2001 to 24% during 2011–2021, and ribbon development also increased from 5% during 1991–2001 to 8% during 2011–2021.

Overall, Lucknow UA exhibited the highest edge expansion urban sprawl typology, followed by Raipur UA, Bhubaneswar UA, and Dehradun UA. Leapfrog development was highest in Lucknow UA, followed by Dehradun UA, Raipur UA, and Bhubaneswar UA. Ribbon development occurred highest in Dehradun UA, followed by Lucknow UA, Raipur UA, and Bhubaneswar UA.

## Discussions

Based on RS and GIS, the spatiotemporal land cover change detection exhibited a widespread built-up land cover growth in all four Indian medium-sized cities selected from the diverse physiographic locations. In Lucknow UA, the rise in built-up during 1991–2001 was prominently due to the development of new institutional buildings, such as Sanjay Gandhi Post Graduate Institute of Medical Sciences, Lucknow University, Lucknow Polytechnic, and Ambedkar Park, and also along the major roads (Shukla & Jain, 2018). Furthermore, during 2001–2011, rapid built-up growth had occurred in the peripheral areas of the north direction primarily due to the conversion of forests and other land covers (Dutta, 2012). Lastly, during 2011–2021, the growth in built-up had occurred in the peripheral areas of north, south, and west direction primarily due to conversion of other land cover. Moreover, Lucknow UA is located in the Indo-Gangetic Plains, wherein the availability of flat surface and fertile soil, combined with the absence of any significant natural barrier had triggered rapid expansion of built-up towards periphery. In Bhubaneswar UA, during 1991–2001, significant built-up growth had occurred in the areas adjoining the old city due to real estate markets as an effect of liberalization of the Indian economy. Furthermore, the rise in built-up during 2001–2011 had occurred due to industries, Software Technology Parks, and further rise in the real estate market (Chettry & Surawar, 2020). Lastly, during 2011–2021, the rise in built-up had extended towards the periphery of the city. Bhubaneswar UA is located in Coastal Plains wherein availability of flat land surface in the peripheral areas combined with the absence of any prominent natural barriers fostered rapid urban growth. The urban growth pattern in Raipur UA during 1991–2001 exhibited a rise in built-up areas surrounding Gol Bazaar, i.e., the central business district (Chettry & Surawar, 2020). Furthermore, during 2001–2011, significant growth in built-up areas was observed due to the declaration of Raipur as a capital city of newly formed Chhattisgarh state of India and industries located in Bhanpuri-Rawabhata and Urla. Lastly, during 2011–2021, the built-up growth accelerated in areas surrounding Atal Nagar (new state capital complex for the state of Chhattisgarh), real estate, and industrial growth within the periphery of the urban agglomeration. In Dehradun UA, the rise in built-up during 1991–2001 was prominently observed in the west direction (Maithani, 2020). Furthermore, during 2001–2011, rapid built-up growth had occurred in the west and south direction due to availability of flat lands and the presence of road network in this region of the city (Maithani, 2020). Lastly, during 2011–2021, the growth in built-up had occurred in the periphery of the south direction. The presence of natural barriers in the northern direction of Dehradun UA has significantly triggered urban growth in the south and west direction.

Comparatively during 1991–2021, Raipur UA exhibited the highest built-up growth rate, i.e., 351% followed by 291% in Lucknow UA, 177% in Dehradun UA, and 174% in Bhubaneswar UA. However, Bhubaneswar UA exhibited the highest land cover occupied by built-up land cover (74%) by the end of the study timeline, i.e., 2021 followed by 68% in Raipur UA, 67% in Dehradun UA, and 60% in Lucknow UA. Overall, a significant rise in built-up land cover was observed, while vegetation and other land cover exhibited a declining trend during the study period in all the selected four cities. A similar trend of rapid urban growth was observed in other cities, such as Ajmer, India (Jat et al., 2008a, b) Dhaka, Bangladesh (Dewan & Yamaguchi, 2009) Kahramanmaras, Turkey (Doygun, 2009) cities in Pearl River Delta, China (Lv et al., 2012) and Ibadan, Nigeria (Adeola Fashae et al., 2020). The rapid conversion of other land covers to built-up areas has caused a rise in urban growth in all the four Indian medium-sized cities. Such transformations were significantly observed in the areas surrounding to the periphery of the selected urban agglomerations.

The major causes of haphazard growth in built-up land cover can be attributed to multiple factors. However, population growth in all the four selected cities has significantly triggered a rise in built-up areas (Dutta et al., 2015; Das et al., 2016; Khan & Jhariya, 2018; Shukla & Jain, 2018). It was observed that the total population in Lucknow UA has increased from 1.66 million in 1991 to 2.90 million in 2011 as per the Census of India 2011. The projected population (average of arithmetic growth method, incremental method, and geometric growth method) for the year 2021 was 3.64 million. Similarly, the total population in Bhubaneswar UA has increased from 0.41 million in 1991 to 0.88 million in 2011 as per the Census of India 2011. The projected population for the year 2021 was 1.17 million. In Raipur UA, the total population has increased from 0.46 million in 1991 to 1.12 million in 2011 as per the Census of India 2011. The projected population for the year 2021 was 1.58 million. The total population in Dehradun UA has increased from 0.36 million in 1991 to 0.70 million in 2011 as per the Census of India 2011. The projected population for the year 2021 was 0.91 million. Moreover, all the four Indian medium-sized cities selected in this study are state capital cities, wherein large-scale infrastructural growth and better quality of life attract migrants from nearby towns and villages. These facilities promote migration from nearby urban areas and villages, and the majority settle in the outskirts due to low housing costs. Such pattern of urban growth was also observed in other cities (Jat et al., 2008a, b; Al-sharif et al., 2017; Hasnine & Rukhsana, 2020). Other factors that might have promoted urban growth include, but are not limited to, lower price of land under vegetation and other land cover categories (agriculture, water body, wetlands, and fallow land), unauthorized or illegal construction in ecologically fragile areas such as forests and water bodies, weak coordination among the UA authorities, and absence of laws which prevents the conversion of agricultural and other natural land covers.

Relative entropy index exhibited the occurrence of urban sprawl in all selected four study areas. Overall, Lucknow UA exhibited high urban sprawl intensity while low urban sprawl intensity was observed in Dehradun UA. Furthermore, Lucknow UA exhibited the highest edge expansion and scatter development typology of urban sprawl, while Dehradun UA showcased the highest ribbon development typology of urban sprawl. Lucknow UA is located in the Indo-Gangetic Plain physiographic region of India wherein it is largely dominated by land with flat surfaces and arable soil; moreover, the absence of natural barriers has encouraged higher urban sprawl and scope for edge expansion and scatter development urban sprawl typologies. Dehradun UA is located in the Northern Mountainous region and is dominated by uneven landforms, difficult topography, and the presence of prominent natural barriers, such as mountain ranges and water bodies discouraged urban sprawl. However, ribbon sprawl was prominently observed in Dehradun UA due to topography and terrain.

The variations in the growth pattern of built-up areas during the study period caused fluctuations in the temporal trend of relative entropy index and urban sprawl typologies. The urban expansion index revealed that edge expansion was the prevalent typology of urban sprawl in all the four study areas. This study validates the observations of Liu et al. (2018) about the significant presence of edge expansion typology of urban sprawl in medium-sized cities. Such development pattern promotes encroachment or unplanned growth in the urban periphery which might get unnoticed or overlooked by the authorities. Thus, urban sprawl has severe implications in the peripheral areas.

In addition, the effects of economic reforms and policies introduced after the liberalization of the Indian economy are also significant. The influx of foreign direct investment (FDI) and the encouragement of the Indian government to establish private sector industries have contributed to industrial growth in and around all four selected study areas. Furthermore, the reform under the Jawaharlal Nehru National Urban Renewal Mission (JNNURM) initiated the abolition of the Urban Land Ceiling Regulation Act (ULCRA) since 2005 and eased the land supply in urban areas. The minimum residential project size for FDI has been reduced to just 30 acres, and 100% of FDI was allowed in the integrated townships and infrastructure projects. Cumulatively, these steps have resulted in real estate and institutional growth in and around the selected cities.

## Conclusion

This research exhibited spatiotemporal urban growth pattern and assessed the urban sprawl in four fast-developing Indian medium-sized cities (Lucknow UA, Bhubaneswar UA, Raipur UA, and Dehradun UA) from 1991 to 2021. It demonstrated the efficient utilization of geospatial measurement techniques in the urban planning field to analyze urban sprawl. The urban growth pattern in the selected cities exhibited a rise in built-up land cover towards the peripheral areas. Raipur UA showcased a higher built-up growth rate compared to other selected cities. There was a significant reduction in vegetation and other land cover categories during the study period. Some of the prominent factors that led to the rapid growth of built-up areas include low price of other land covers, haphazard and unchecked urban growth, and weak coordination among the multiple authorities of UA. The relative entropy index detected the prevalence of urban sprawl in all four cities. Moreover, the trend indicated a continuous rise in urban sprawl from 1991 to 2021 with varying intensities. Edge expansion was the most prevalent typology of urban sprawl in all four cities, followed by leapfrog development and ribbon development. Overall, economic reforms and policies enacted after liberalization did not seek to curb urban sprawl. Instead, it encouraged indiscriminate city growth. Therefore, there is an urgent need for sustainable urban planning in Indian medium-sized cities to promote sustainable cities and communities.

Urban sprawl is a complex and dynamic phenomenon; therefore, this study combined RS and GIS with the entropy index and urban expansion index. The methodology adopted in this study can be used to assess the urban sprawl in other medium-sized cities located in India and other countries due to its importance in the coming decades. The obtained results would guide planners and authorities to prepare relevant strategies for promoting urban sustenance. The draft National Land Utilization Policy prepared for protecting agricultural lands from rapid and unplanned land cover conversions should be urgently finalized and implemented. Smart growth concepts such as infill development, increased FSI, mixed-use development, transit-oriented development, conservation of open spaces and forests, and community participation shall be promoted through the urban development plans. The availability of high-resolution satellite imagery and socio-economic data for the selected cities remains a major limitation of this study. The future scope of this study is to use machine learning tools to predict changes in land cover in all selected cities and further analyze urban sprawl phenomena by gathering datasets, such as socio-economic conditions, accessibility, and economy.

## Data Availability

Data will be made available upon reasonable request.
